# Supplementary material for: From pixels to connections: exploring in vitro neuron reconstruction software for network graph generation
Source: Commun Biol. 2024 May 15;7:571. doi: 10.1038/s42003-024-06264-9 (PMC11096190; doi:10.1038/s42003-024-06264-9)
Supplement: Supplementary file 2 — Supplemental Material [file 42003_2024_6264_MOESM2_ESM.pdf]

Supplementary Material

Table of Contents

GLOSSARY OF COMPUTATIONAL TERMS RELEVANT TO BIOIMAGE ANALYSIS. 2

SUPPLEMENTARY FIGURE 1. PRE-PROCESSING. 6

SUPPLEMENTARY FIGURE 2. SEGMENTATION. 7

SUPPLEMENTARY FIGURE 3. MORPHOLOGICAL LABELLING 8

SUPPLEMENTARY FIGURE 4. POST-PROCESSING. 9

SUPPLEMENTARY FIGURE 5. NETWORK RECONSTRUCTION. 9

Glossary of computational terms relevant to bioimage analysis

Adaptive region growing: A local approach to image segmentation. Starting from seed points, segmented regions are established by merging neighboring pixels if they display similarity in characteristics such as intensity or colour. The threshold driving this process dynamically changes to accommodate new contextual information in the regional neighborhood.

Adaptive thresholding: A technique to separate objects of interest from background by calculating and applying different threshold values in local pixel neighborhoods. This method shows better adaption to local image variations than global thresholding, and thus may perform better in images with uneven illumination or varying contrast.

Attachment point: A point in a neurite skeleton that marks its attachment to a somata. This may represent the location at which an outgoing neurite has sprouted from the cell, or an ingoing neurite has made contact with the cell.

Bayesian framework: A statistical approach that relies on prior knowledge about a situation to update and refine that knowledge based on observed evidence.

Bottom-hat filtering: A filter that enhances dark structures on a bright background. Morphological closing is performed on the image, and the original image is subtracted from this result.

Branch point: A point in a neurite skeleton where multiple branches converge. This may be due to a single neurite bifurcating into multiple branches, or an intersection or crossover of numerous neurites.

Convolutional neural network (CNN): A deep learning algorithm predominantly used in computer vision pipelines to detect and differentiate image objects. The core CNN architecture is comprised of artificial neurons that self-optimize by learning to extract increasingly complex and high-level features. For example, this hierarchical pattern recognition could start by detecting edges and curves, combining these to form shapes such as circles, and finally combining these to form segmented cell somata.

Difference of Gaussians filter: a method to enhance the edges in an image, whereby a Gaussian blurred version of the image is subtracted from a less blurred version.

End point: A point in a neurite skeleton that marks the terminal of a neurite.

Euclidean distance: The distance between two points with Cartesian coordinates.

Frangi's filter[1]: A filter that identifies and enhances filamentous structures based on local geometric properties while suppressing background noise.

Gaussian blur: A transform that reduces detail and noise by blurring an image with a Gaussian function. This is classified as a low pass filter as high frequency components are attenuated.

Hidden Markov Model: A probabilistic model that infers future unknown states using information from previously observed states.

Intensity threshold (global): A defined pixel intensity range that is used to distinguish foreground from background pixels in binarization. This technique is highly useful for segmenting objects where the intensity of structures of interest are strongly distinct from extraneous signal.

Greyscale Morphology filter[2]: An ImageJ filter that performs morphological operations on greyscale images, available at https://imagej.nih.gov/ij/plugins/gray-morphology.html.

Hysteresis linking technique: To connect fragmented segments, this technique initially uses thresholding to detect strong candidate pixels in the space between segments. These candidates are then linked into a continuous structure by detecting the path of highest gradient magnitude where the strongest edge most likely exists.

Laplacian filter: A method that identifies the regions of the image that have a rapid change in intensity consistent with the presence of an object edge. To achieve this, a second-order differential operator is used to measure the gradient of intensity function. This process is highly sensitive to noise and for this reason is often paired with a Gaussian smoothing prior.

Laplacian high pass filter: A filter that enhances high-frequency components or edges while suppressing low-frequency components.

Level set method: an approach to detect the shape and edges of complex objects. A mathematical function is progressively deformed to the object using a cost minimization function. Ideally this process achieves a balance between data-fitting and regularization.

Median filter: A blurring technique to reduce noise. The filter calculates the median value of a pixel neighborhood and sets this as the pixel value.

Morphological closing: A mathematical operation that is performed on an image to fill small gaps or holes. It is achieved by dilating and eroding the segmentation using the same structuring element.

Morphological opening: A mathematical operation that is performed on an image to remove small foreground objects. It is achieved by eroding and dilating the segmentation using the same structuring element.

Niblack thresholding[3]: An adaptive thresholding technique based on the mean and standard deviation of pixel neighborhoods.

Non-maximum suppression: A technique to refine object identification by suppressing redundant or inappropriate signals. If the composition of a structure is ambiguous, this approach selects a set of detection candidates and compares their confidence scores or probabilities to determine which subset is most likely part of the structure.

Otsu thresholding[4]: An automatic thresholding technique that selects an optimal threshold value based the minimization of intra-class intensity variance.

OpenCV: a library of cross-platform computer vision functions available at https://opencv.org/.

Per Object Ellipse fit method[5]: A segmentation method that uses ellipse fit to optimize the threshold for each object based on shape and size. Although this method was originally designed for circular objects, an adjustment of parameters allows the detection of elongated structures such as neurons.

Rolling ball background subtraction: A background subtraction technique that also corrects uneven illumination. A local value is determined by averaging a large ball-shaped group around each pixel and subtracting this from the original image.

Skel2Graph[6]: A Matlab function that converts a skeletonized filamentous structure into a network graph with nodes as branch and end points.

SKimage: a library of image processing functions for Python available at https://scikit-image.org/.

Sobel filter: A technique commonly used for edge detection and noise reduction based on a first-order derivative operator. This filter maximally detects edges running vertically and horizontally to the gradient of the image.

Stochastic completion field: A mathematical model that estimates the shapes that most likely complete partial object boundaries with the overall objective of forming a cohesive structure. This estimation is iteratively updated based on characteristics of surrounding pixels in multiple domains such as intensity or texture. The resulting probability distribution informs the selection of optimal candidate choices.

Support Vector Machine: A supervised machine learning approach used for classification and regression tasks.

Top-hat filtering: A filter that corrects uneven illumination in images with a dark background. With a kernel shaped like a top hat, the morphological opening of the image is calculated and the result is subtracted from the original image.

Transfer entropy: A statistic in information theory that establishes causal relationships between variables in complex processes across time. This is achieved by defining the extent to which the past state of a variable informs the future state of another variable outside of what can be predicted by its historical behavior.

W-Net: A deep learning algorithm for unsupervised image segmentation. The architecture includes an encoder that establishes the segmentation, as well as a decoder that builds an image from this segmentation.

Watershed algorithm: A technique particularly suited to the segmentation of circular objects that are touching or overlapping. The image is conceptualized as a topographic surface with peaks (local maxima) and valleys (local minima). In the analogy of "flooding" this landscape, valleys and surrounding catchment basins fill with water and the boundary where the water from one catchment meets another are recognized as the edges of objects in the resulting segmentation.

Weiner filter[7]: A pre-processing technique that reduces noise and preserves signal. This is achieved by leveraging the statistical properties of the signal and noise to restore an approximation of the original content.

Zhang-Suen thinning algorithm[8]: A skeletonization algorithm that iteratively applies a set of rules to remove pixels from a segmentation, eventually thinning it to a one pixel- thick representation that preserves connectivity.

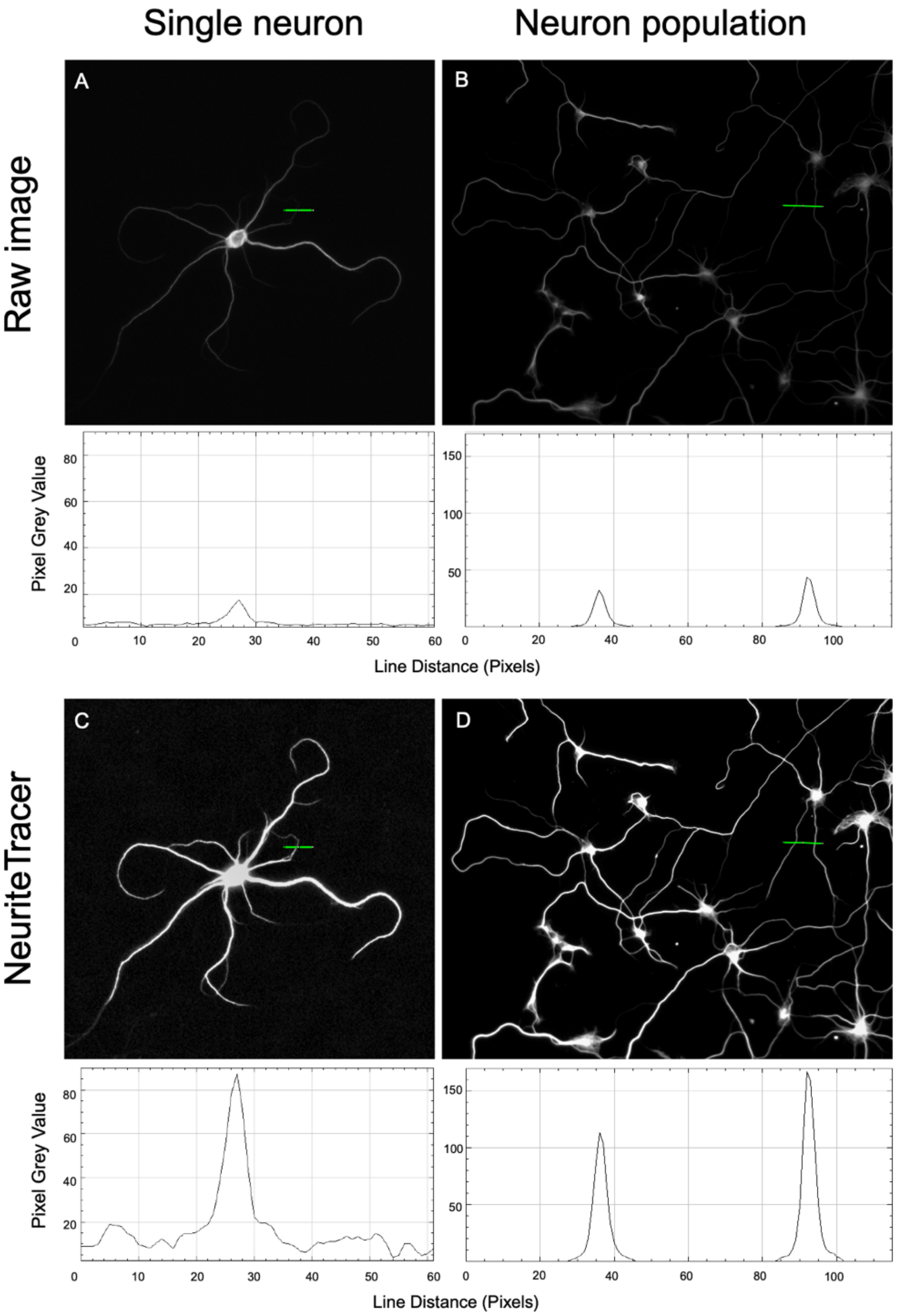

Supplementary Figure 1. Pre-processing. Pre-processing improves the signal of target biological structures in microscopy images. **(A,B)** Raw images sourced from Cell Image Library[9,10] with pixel grey values along green line ROIs plotted in graphs below. **(C,D)** Corresponding pre-processed outputs performed by NeuriteTracer[11], which applies contrast enhancement, rolling ball radius background subtraction, despeckling and Gaussian blurring. Notable improvements in neuron structure signal are observed in pixel grey value plots below.

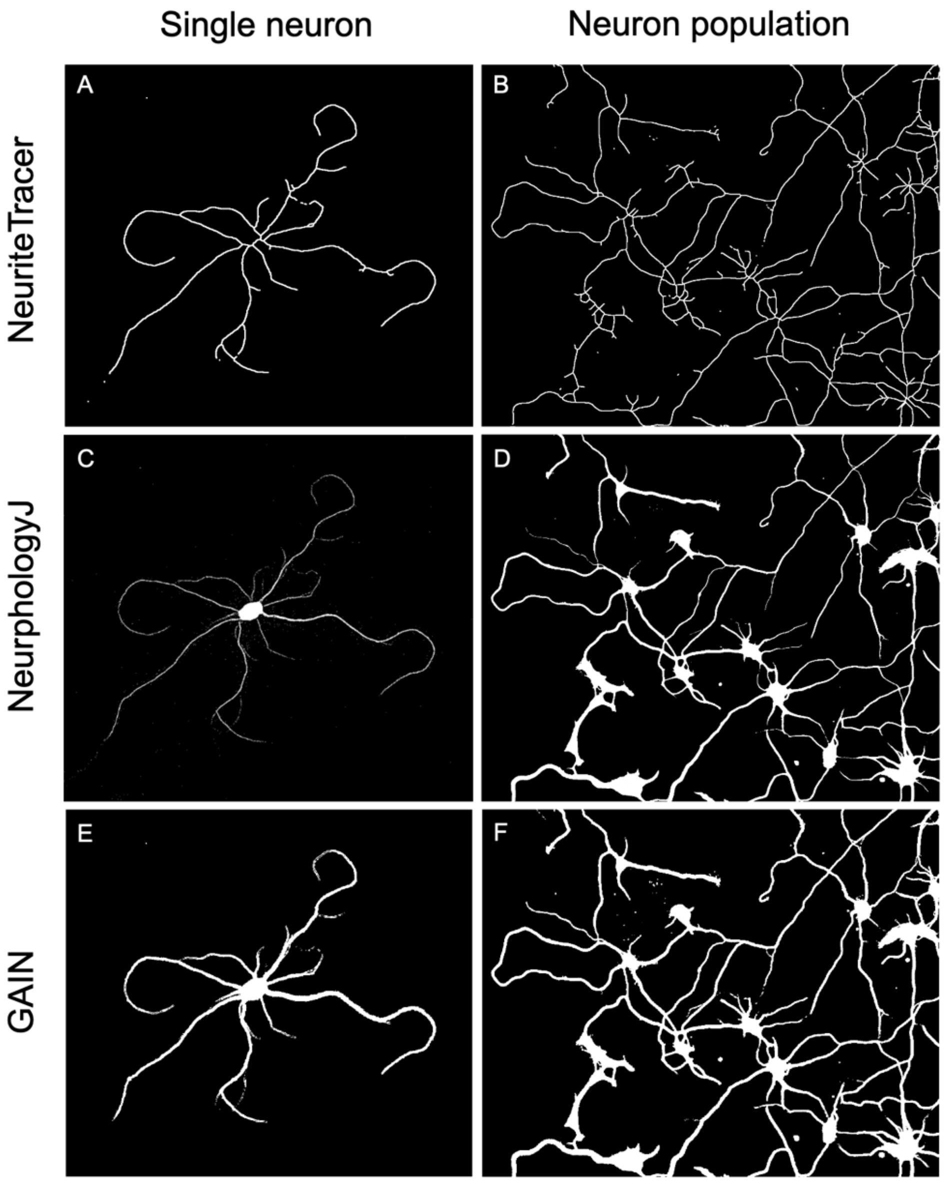

Supplementary Figure 2. Segmentation. Segmentation outputs generated by three neuron reconstruction tools on two microscopy images[9,10]. **(A,B)** NeuriteTracer[11] achieves a binarized image via global thresholding, then performs skeletonization. **(C,D)** NeurphologyJ[12] performs segmentation using a combination of edge detection and global thresholding. **(E,F)** GAIN[13] achieves segmentation through edge detection and double Otsu thresholding. (NeurphologyJ and GAIN images recoloured for visualization.)

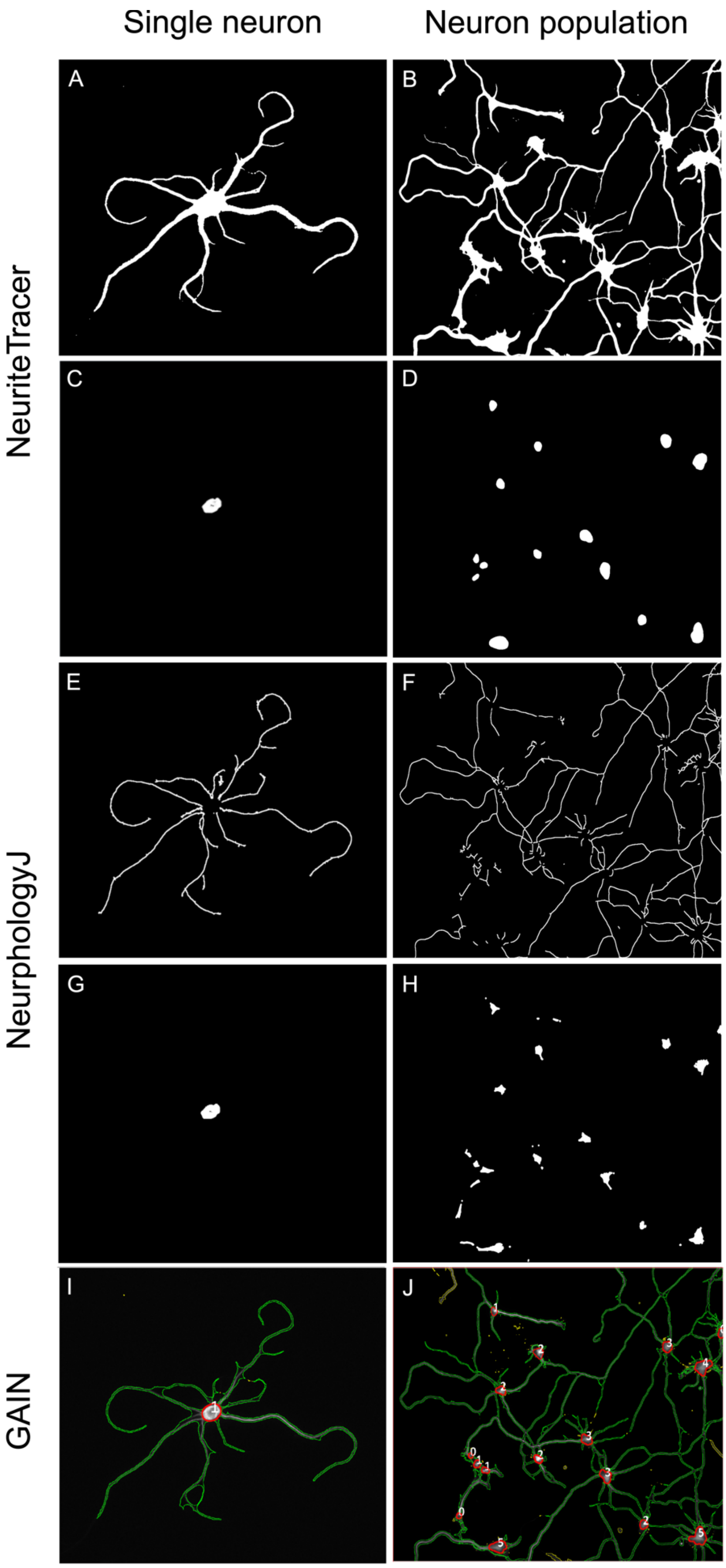

Supplementary Figure 3. Morphological Labelling. Software-generated morphological labels for microscopy images. NeuriteTracer[11] segmentation of neurons **(A,B)** and somata **(C,D)** achieved through global thresholding of a neuron-nuclei image pair. NeurphologyJ[12] segmentation of neurites **(E,F)** and somata **(G,H)** achieved through a series of morphological operations including opening (erosion followed by dilation). **(I,J)** GAIN[13] segmentation of neurons (green boundaries) and nuclei (red boundaries), achieved through edge detection and thresholding a neuron-nuclei image pair. (NeurphologyJ and GAIN output recoloured and dilated for visualization. See footnote*.)

* As nuclei-stained image was not available for Cell Image Library dataset[9,10], synthetic nuclei images were generated and used as inputs for NeuriteTracer and GAIN. Nuclei ROIs were manually traced, masked, and overlaid on neuron images with morphological AND operator.

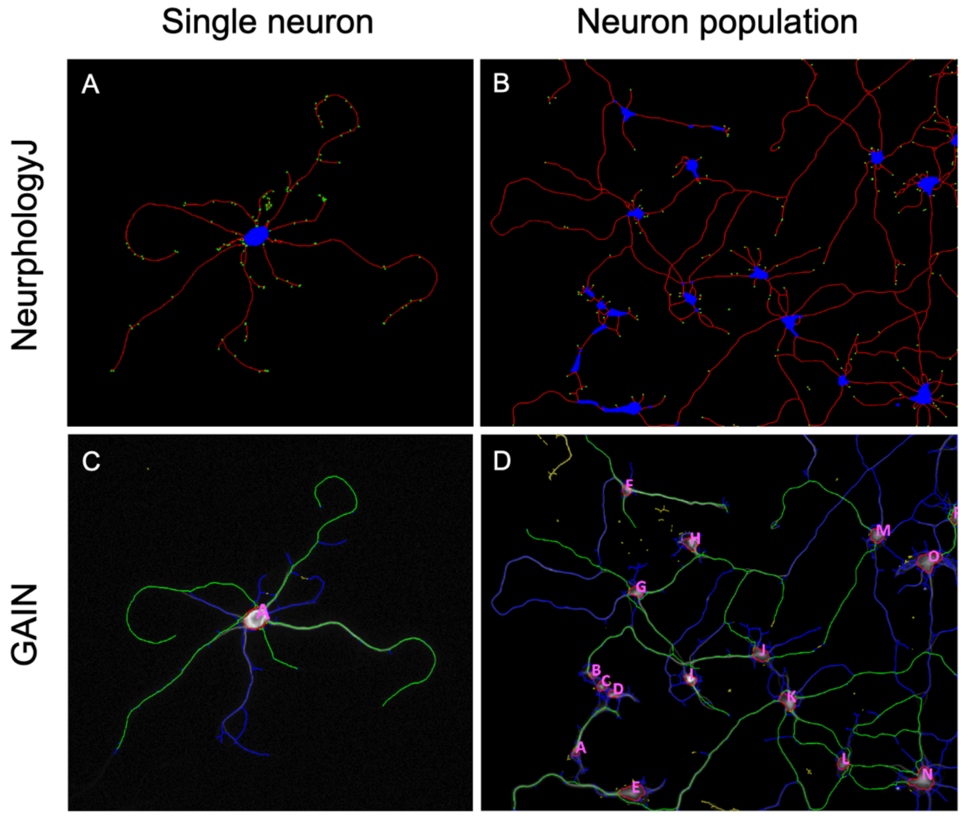

Supplementary Figure 4. Post-processing. (A,B) NeurphologyJ[12] produces reconstructions comprised of somata (blue), skeletonized neurites (red), as well as end-, attachment- and branch-points (green). **(C,D)** GAIN[13] individuates primary neurites, including long neurites which have a length more than three times the parent somata (green), short neurites (blue), as well as neurites that are not connected to a somata (yellow).

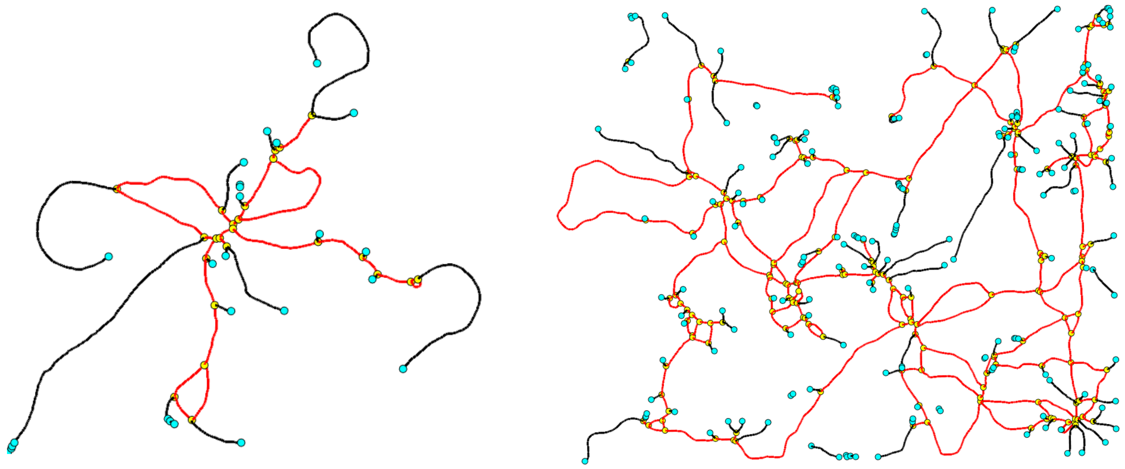

Supplementary Figure 5. Network reconstruction. Graphs generated with the Skel2Graph3D[6] algorithm using NeuriteTracer[11] tracings (Supplementary Figure 2a,b) as input. Connectivity is based on neurite skeleton properties rather than cell body localization. Cyan nodes depict end points, yellow nodes depict branch points, red connections depict edges, and black connections depict terminal edges.

Supplementary References

1. Frangi, A., Niessen, W., Vincken, K. & Viergever, M. Multiscale vessel enhancement filtering Medical Image Computing and Computer-Assisted Interventation — MICCAI'98. in *Medical Image Computing and Computer-Assisted Interventation — MICCAI'98* vol. 1496 (1998).

2. Prodanov, D. & Verstreke, K. Automated Segmentation and Morphometry of Cell and Tissue Structures. Selected Algorithms in ImageJ. in *Molecular Imaging* (2012). doi:10.5772/36729.

3. Niblack, W. An introduction to digital image processing. *An introduction to digital image processing.* (1986) doi:10.1007/978-3-030-88439-0_14.

4. Otsu, N. THRESHOLD SELECTION METHOD FROM GRAY-LEVEL HISTOGRAMS. *IEEE Trans Syst Man Cybern* **SMC-9**, (1979).

5. Ranefall, P., Sadanandan, S. K. & Wahlby, C. Fast adaptive local thresholding based on ellipse fit. in *Proceedings - International Symposium on Biomedical Imaging* vols. 2016-June (2016).

6. Kollmannsberger, P. *et al.* The small world of osteocytes: Connectomics of the lacuno-canalicular network in bone. *New J Phys* **19**, (2017).

7. Wiener, N. *Extrapolation, Interpolation, and Smoothing of Stationary Time Series*. *Extrapolation, Interpolation, and Smoothing of Stationary Time Series* (2019). doi:10.7551/mitpress/2946.001.0001.

8. Cheriet, M., Kharma, N., Liu, C. L. & Suen, C. Y. *Character Recognition Systems: A Guide for Students and Practioners*. *Character Recognition Systems: A Guide for Students and Practioners* (2007). doi:10.1002/9780470176535.

9. Withers, G. CIL:12434, Rattus, multipolar neuron. *CIL* (2011) doi:https://doi.org/doi:10.7295/W9CIL12434.

10. Brandner, D. & Withers, G. CIL:10111, Rattus, multipolar neuron. *CIL* (2010) doi:https://doi.org/doi:10.7295/W9CIL10111.

11. Pool, M., Thiemann, J., Bar-Or, A. & Fournier, A. E. NeuriteTracer: A novel ImageJ plugin for automated quantification of neurite outgrowth. *J Neurosci Methods* **168**, (2008).

12. Ho, S. Y. *et al.* NeurphologyJ: An automatic neuronal morphology quantification method and its application in pharmacological discovery. *BMC Bioinformatics* **12**, (2011).

13. Long, B. L. *et al.* GAIN: A graphical method to automatically analyze individual neurite outgrowth. *J Neurosci Methods* **283**, (2017).
